# Supplementary material for: Electrical resistance of the current collector controls lithium morphology
Source: Nat Commun. 2022 Jul 9;13:3986. doi: 10.1038/s41467-022-31507-w (PMC9276694; doi:10.1038/s41467-022-31507-w)
Supplement: Supplementary file 1 — Supplementary Information [file 41467_2022_31507_MOESM1_ESM.docx]

**Supplementary Information**

**Electrical Resistance of the Current Collector Controls Lithium Morphology**

Solomon T. Oyakhire^1ǂ^, Wenbo Zhang^2ǂ^, Andrew Shin^2^, Rong Xu^2^, David T. Boyle^3^, Zhiao Yu^3^, Yusheng Ye^2^, Yufei Yang^2^, James A. Raiford^1^, William Huang^2^, Joel R. Schneider^1^, Yi Cui^2,4*^, Stacey F. Bent^1*^

1 Department of Chemical Engineering, Stanford University, Stanford, CA 94305, USA.

2 Department of Materials Science and Engineering, Stanford University, Stanford, CA 94305, USA.

3 Department of Chemistry, Stanford University, Stanford, CA 94305, USA.

4 Stanford Institute for Materials and Energy Sciences, SLAC National Accelerator Laboratory, 2575 Sand Hill Road, Menlo Park, CA 94025, USA.

^ǂ^ These authors contributed equally to this work.

* Email: yicui@stanford.edu

* Email: sbent@stanford.edu


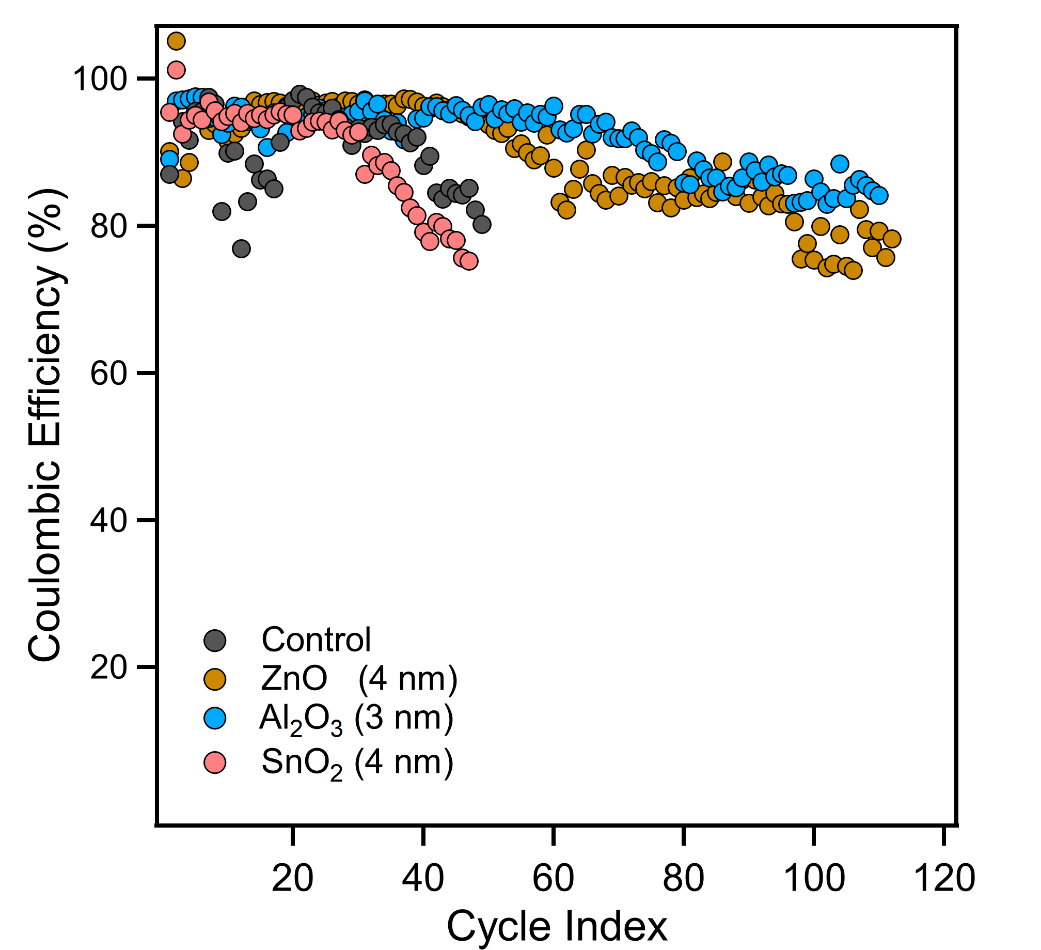


**Supplementary Figure 1.** Coulombic efficiency of Li/Cu cells cycled with DDN electrolyte using bare copper (control) and copper modified with ~4 nm of ALD films.


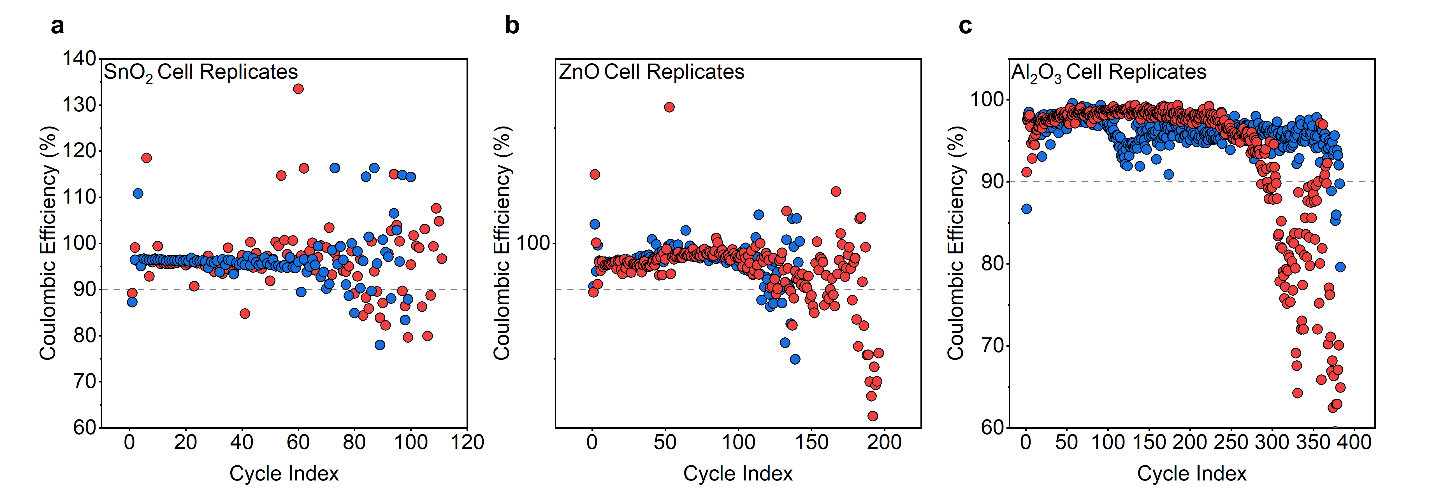


**Supplementary Figure 2.** CE of replicate Li/Cu cells cycled at 1 mA/cm^2^ using copper modified with 7-8 nm of a) SnO_2_, b) ZnO, and c) Al_2_O_3_. Red and blue markers represent separate cell measurements.


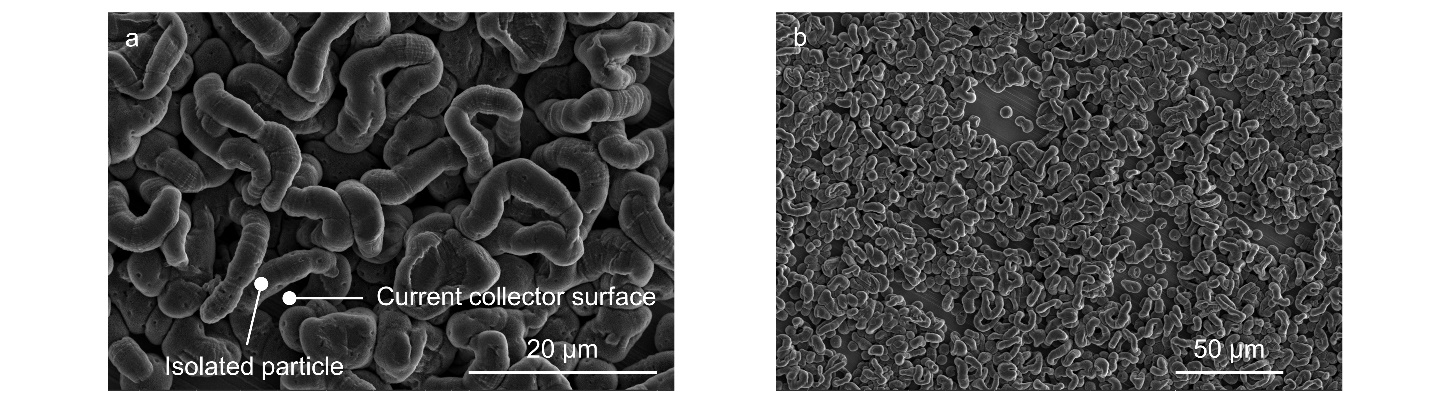


**Supplementary Figure 3.** Li particles deposited on 7 nm of SnO_2_ shown in a) High magnification , and b) Low magnification.


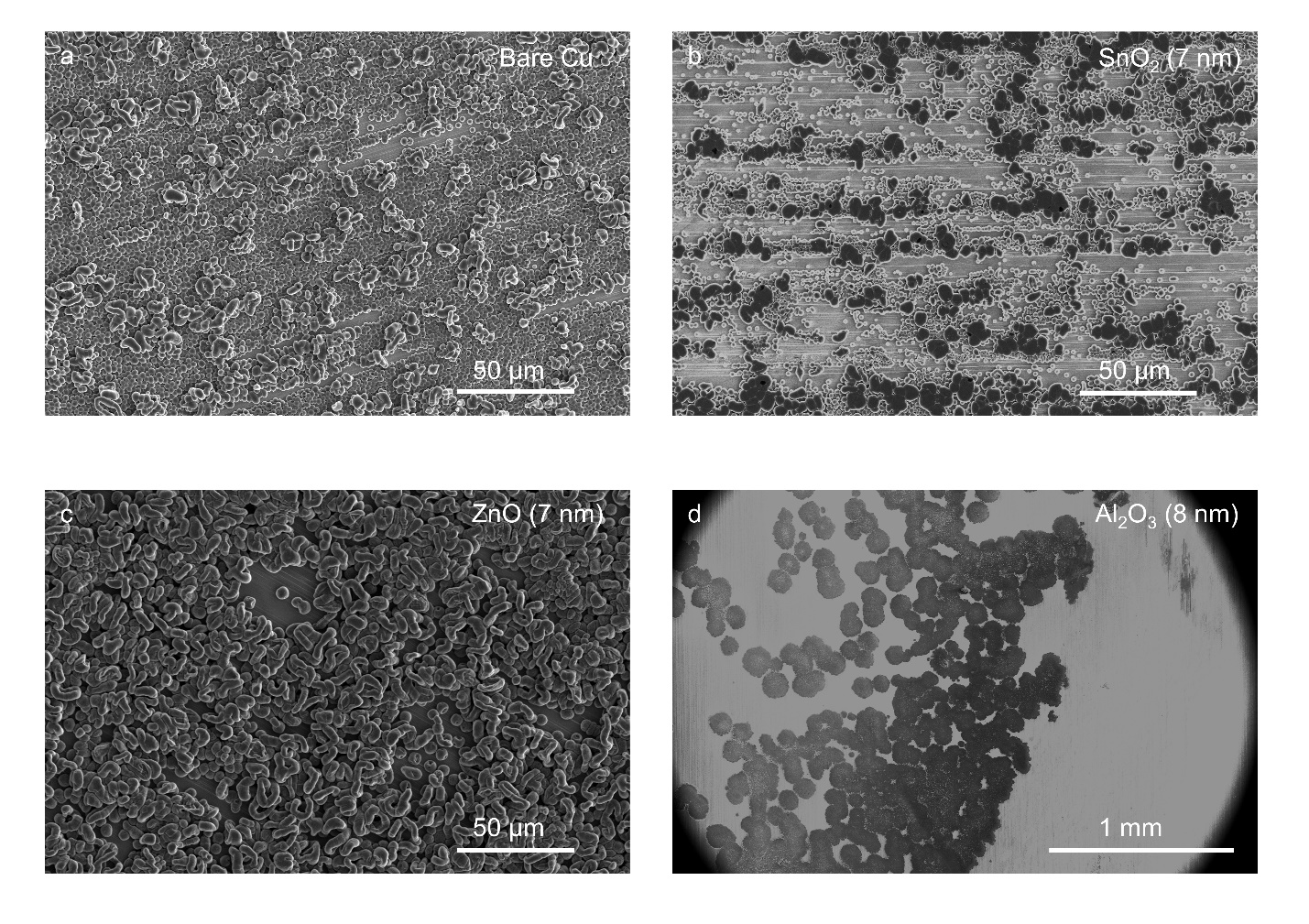


**Supplementary Figure 4.** SEM images revealing representative Li particles across the a) Bare Cu, b) SnO_2_, c) ZnO, and d) Al_2_O_3_ substrates respectively.


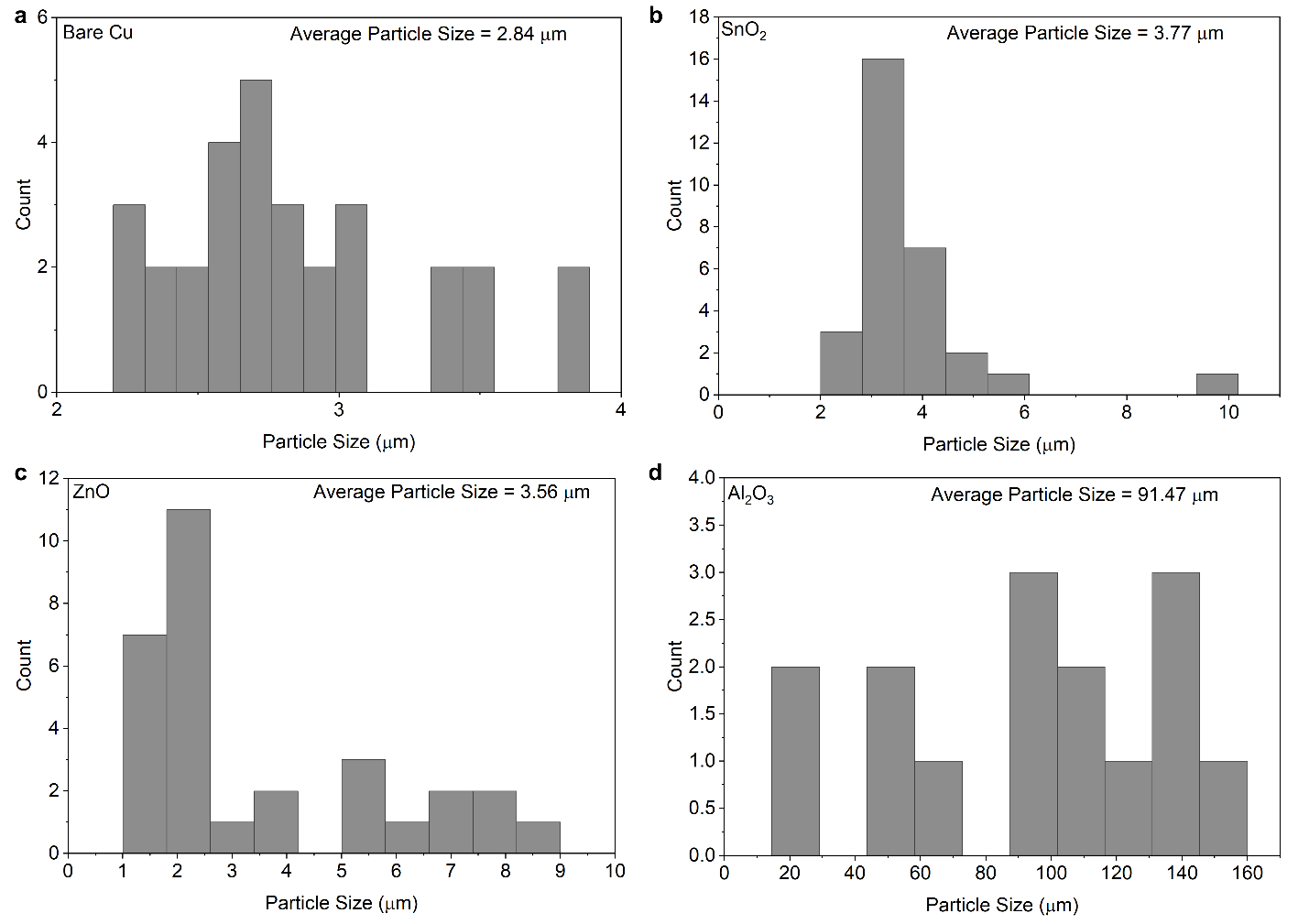


**Supplementary Figure 5.** Particle size distribution analysis for Li deposited on a) Bare Cu, b) SnO_2_, c) ZnO, and d) Al_2_O_3_ substrates respectively.


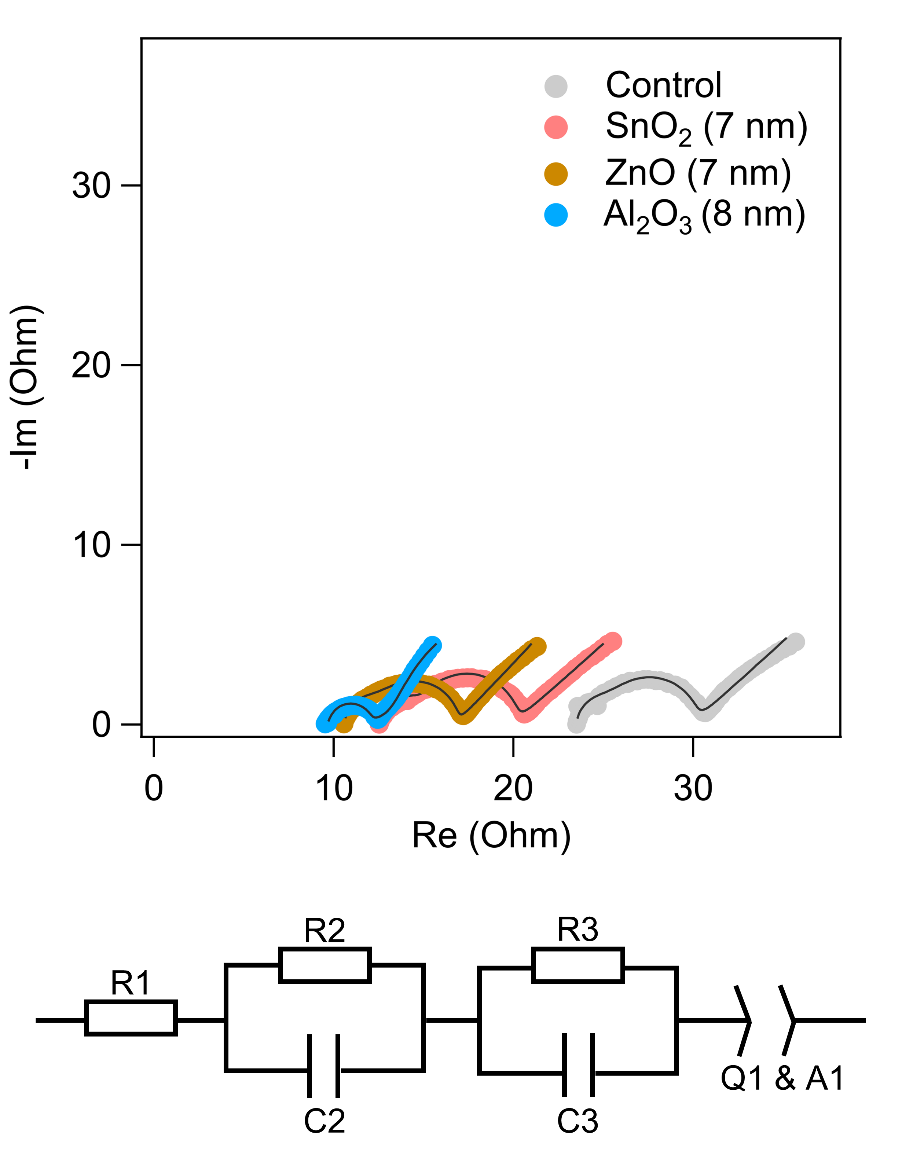


**Supplementary Figure 6.** Electrochemical impedance of Li deposited after 50 electrochemical cycles using bare copper and copper modified with ~8 nm of ALD films. Solid black lines represent EIS fits obtained using the equivalent circuit at the bottom of the figure. Parameters obtained from the fits are presented in supplementary table 1.


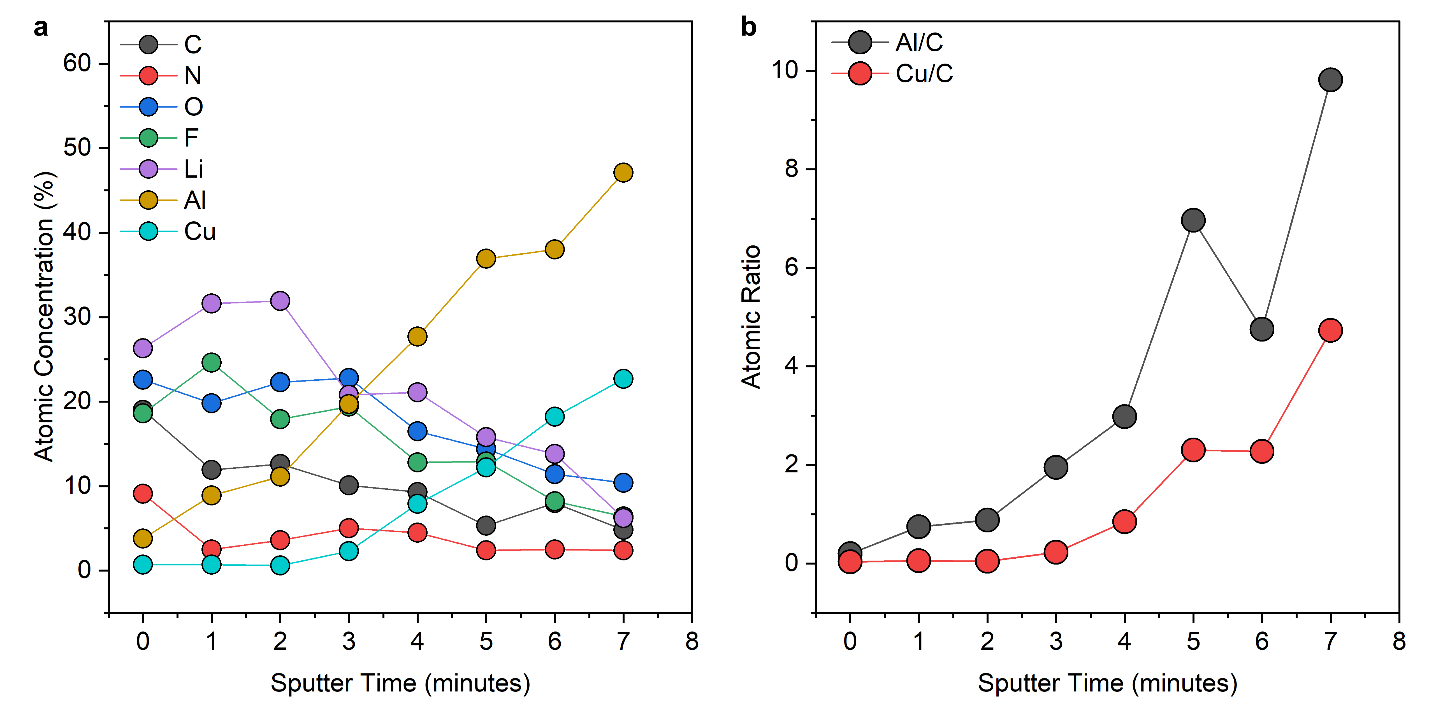


**Supplementary Figure 7.** Sputter profile of 0.1 mAh/cm^2^ of Li, sputtered at 2 nm/min calibrated for SiO_2_. a) Atomic concentration as a function sputter time. b) Atomic ratio as a function of sputter time.


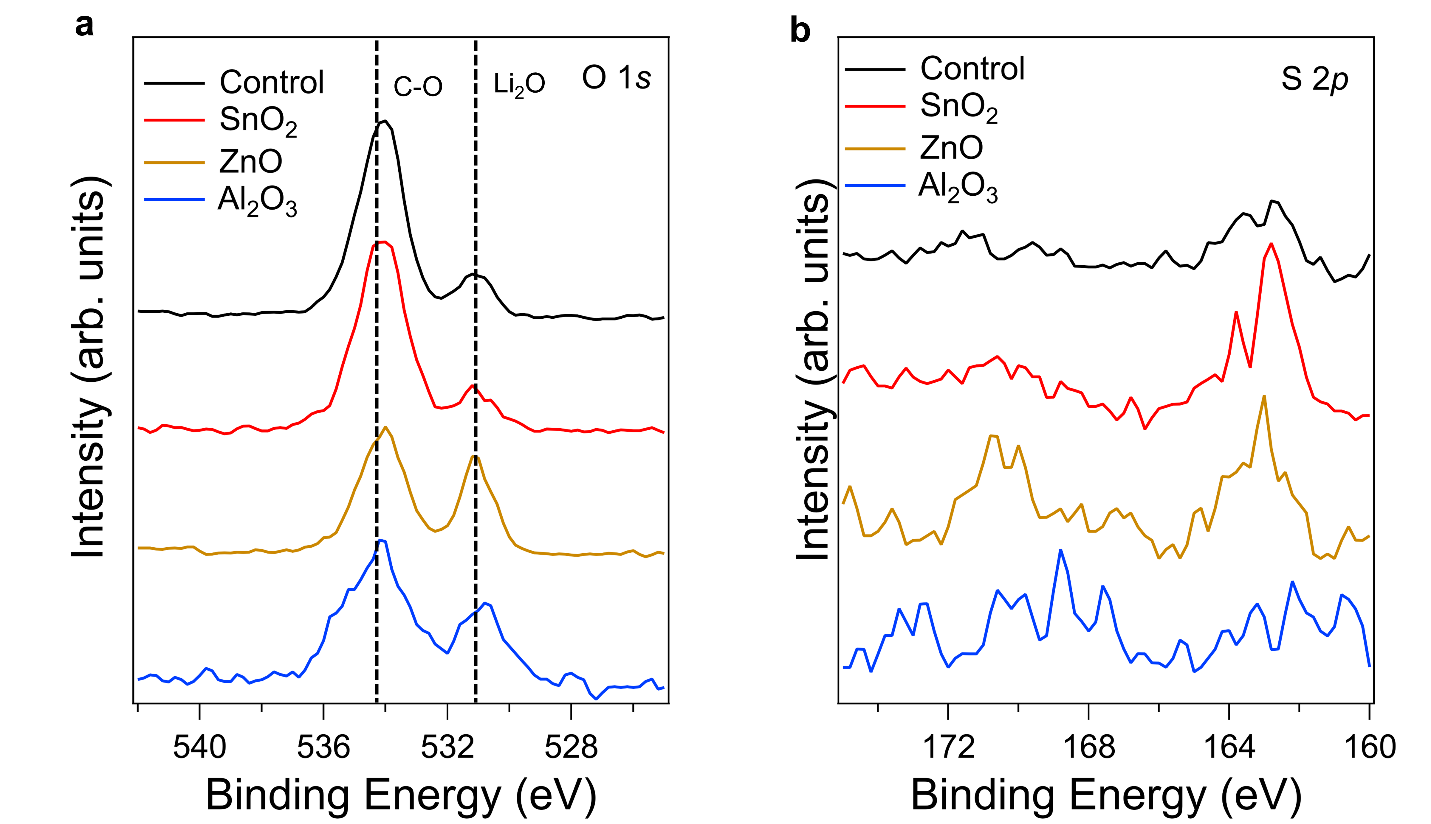


**Supplementary Figure 8.** XPS high resolution scan of SEI formed on lithium deposited atop bare copper and ALD modified copper. **a)** O 1s signals. **b)** S 2p signals.


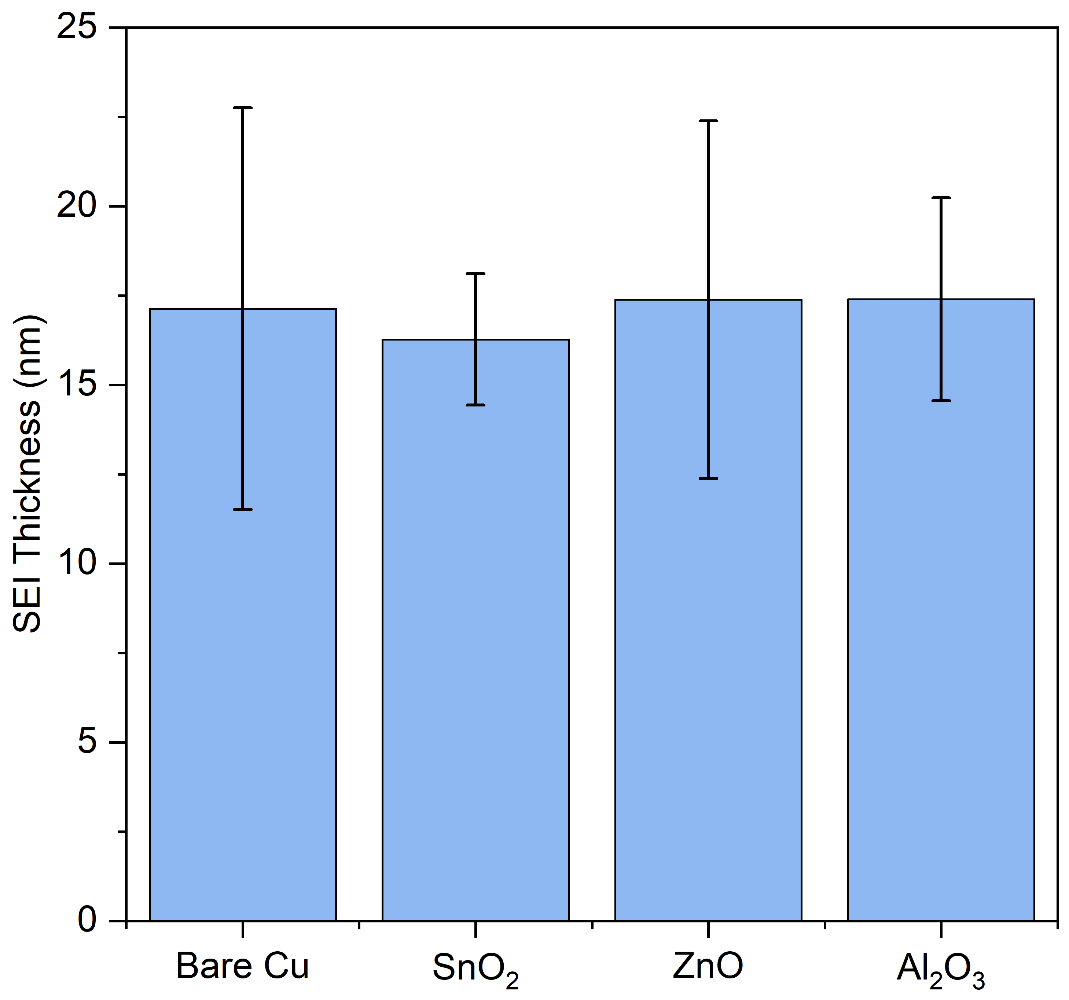


**Supplementary Figure 9.** SEI thickness averaged over 10 distinct locations on a lithium particle deposited on the corresponding substrates, with each error bar representing one standard deviation from the average.


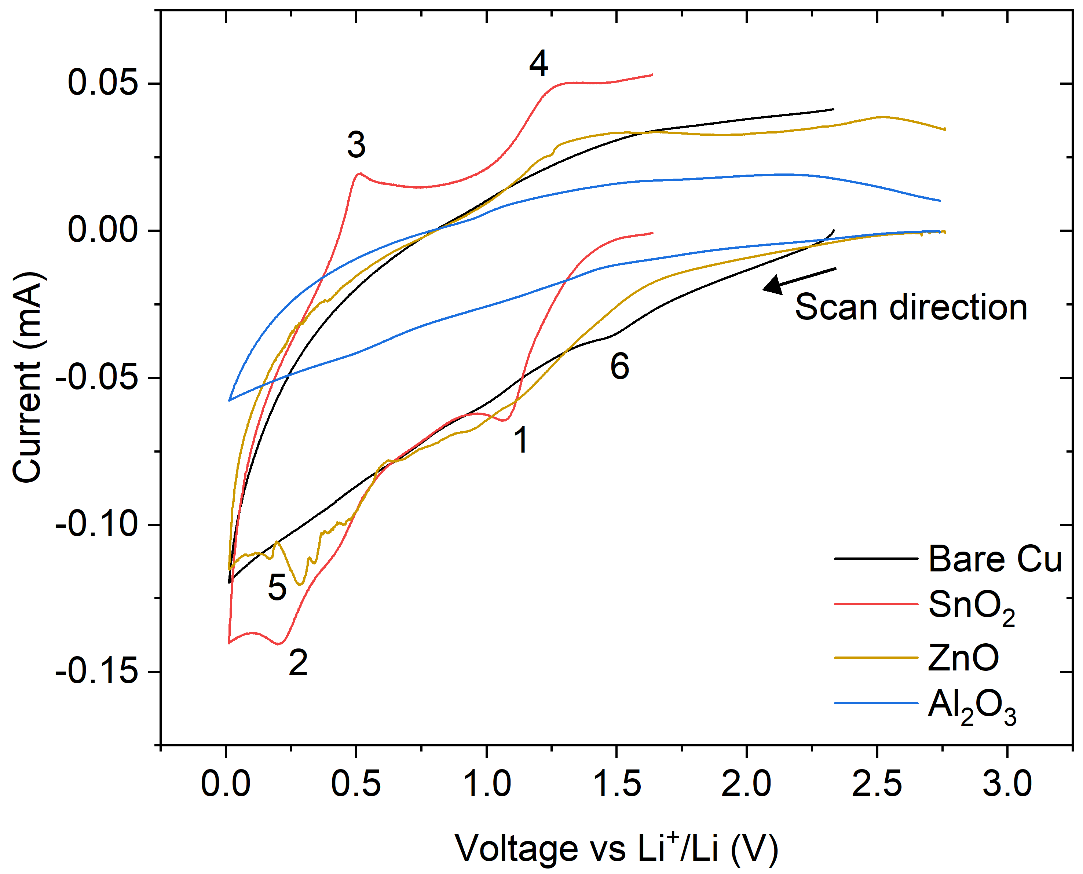


**Supplementary Figure 10.** Cyclic voltammetry scans between OCV and 15 mV at 1 mv s^-1^ carried out for all substrates using DDN electrolyte. Points 1,2,3, and 4 correspond to cathodic and anodic currents associated with the conversion and alloy reactions of SnO_2_.^1,2^ Point 5 corresponds to Li and ZnO alloy reactions.^3^ Point 6 corresponds to conversion reactions between Li and CuO.^4^


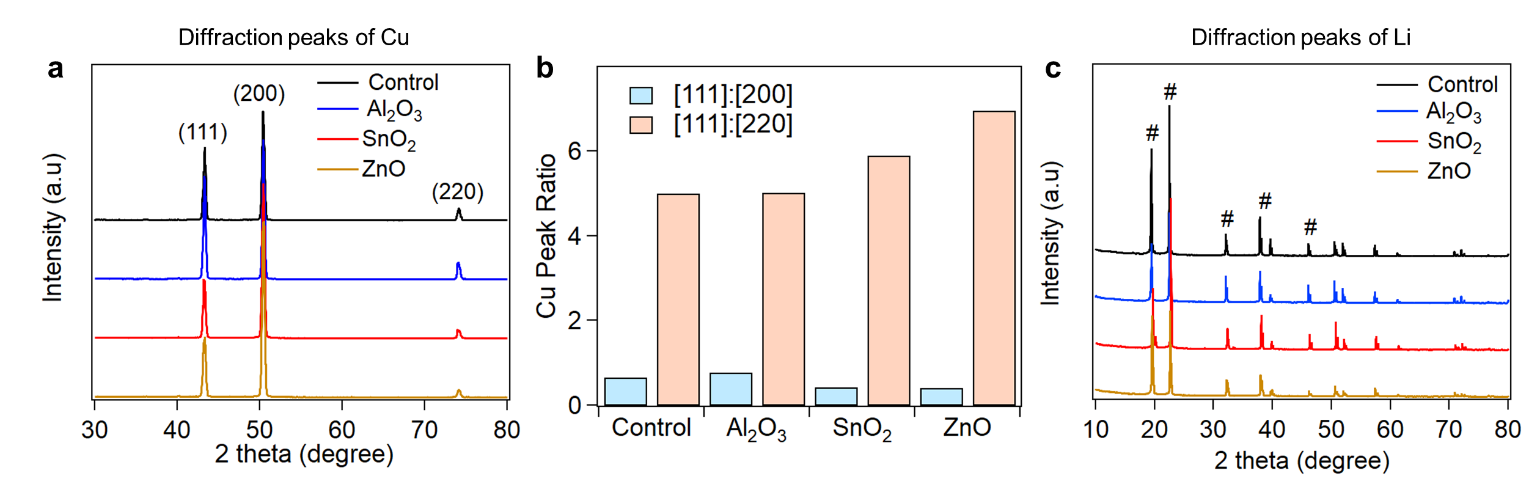


**Supplementary Figure 11.** **a)** XRD analysis of copper substrate before and after ALD modifications. **b)** Integrated facet ratio of Cu XRD peaks in bare and ALD-modified copper. **c)** XRD analysis of Li plated on bare copper and ALD-modified copper; # indicates characteristic lithium peaks.


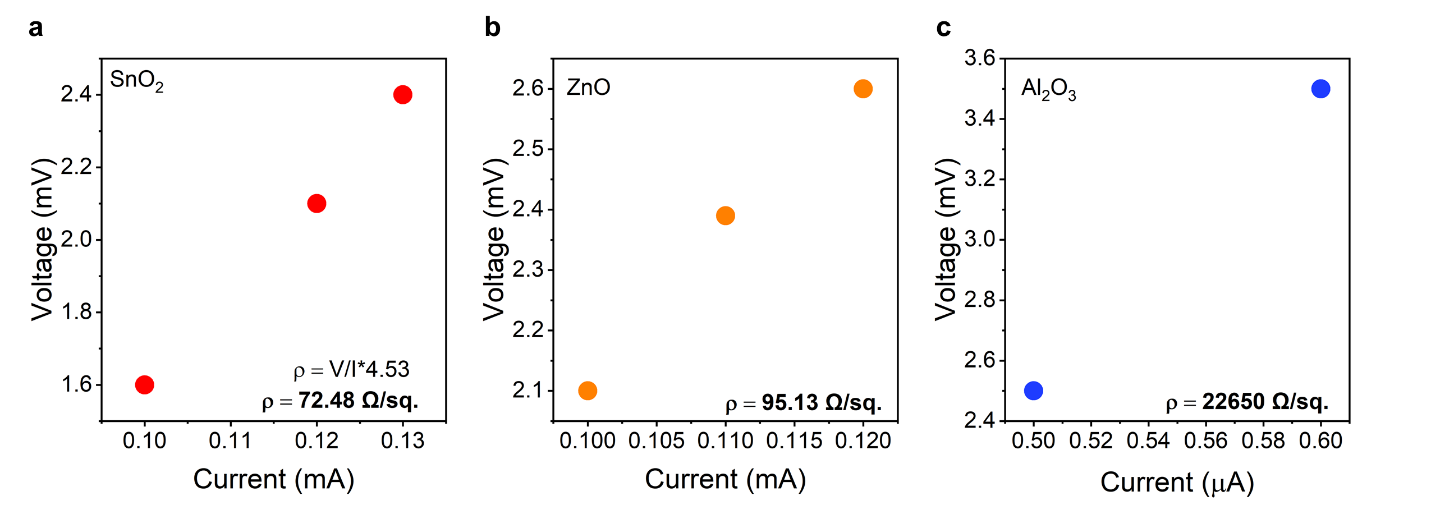


**Supplementary Figure 12.** Electrical resistivity measurements for Si wafers modified with ALD films. I-V relationships for 50 nm films of **a)** SnO_2_, **b)** ZnO, and **c)** Al_2_O_3_. Extracted resistivity values are shown on each figure. The constant, 4.53, is a geometric factor that accounts for thin sheets.


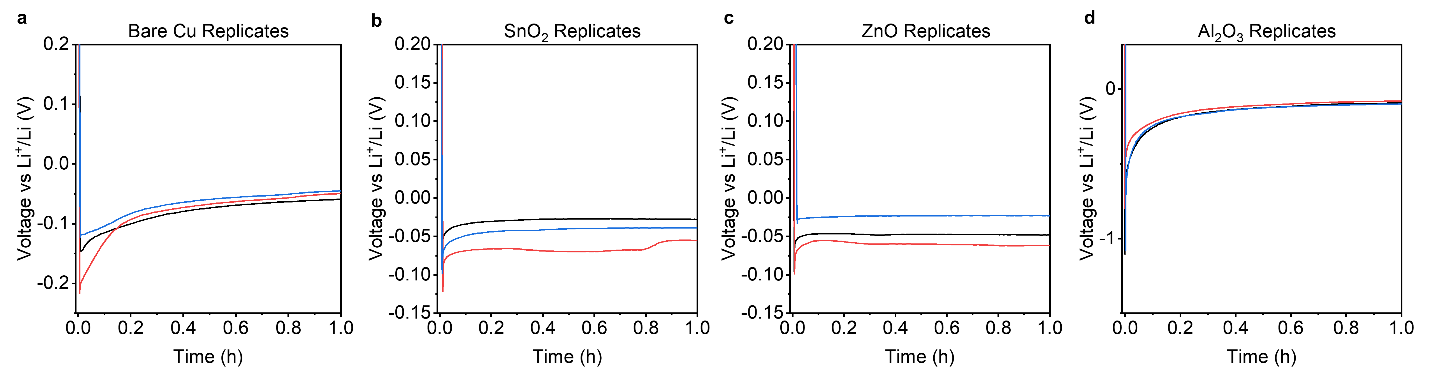
**Supplementary Figure 13.** Voltage profiles showing the first cycle of lithium deposition at 1 mA/cm^2^ on a) bare copper, and ~ 8 nm films of **b)** SnO_2_, **c)** ZnO, and **d)** Al_2_O_3_, showing three cells each.


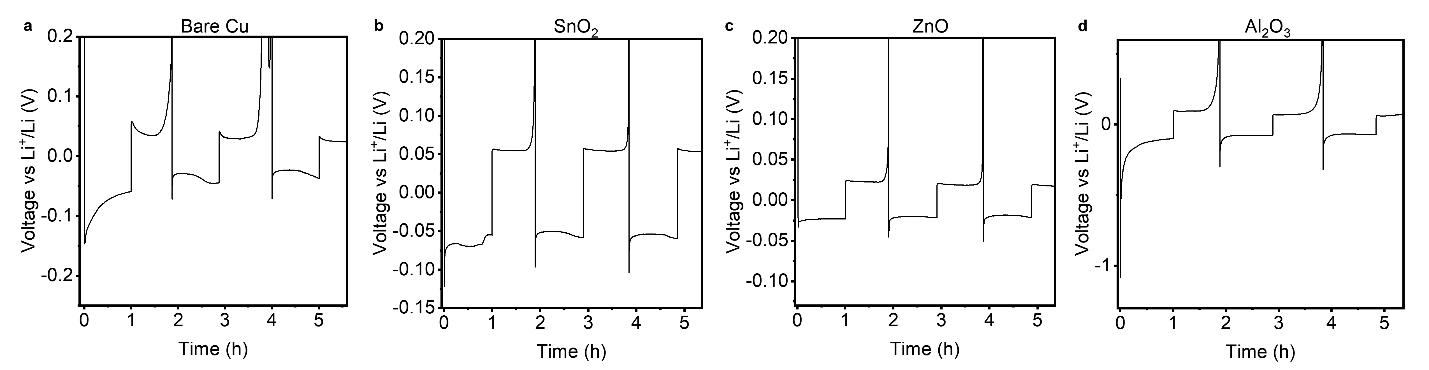


**Supplementary Figure 14.** Voltage profiles showing the first three cycles of lithium deposition at 1 mA/cm^2^ on a) bare copper, and ~ 8 nm films of **b)** SnO_2_, **c)** ZnO, and **d)** Al_2_O_3_.


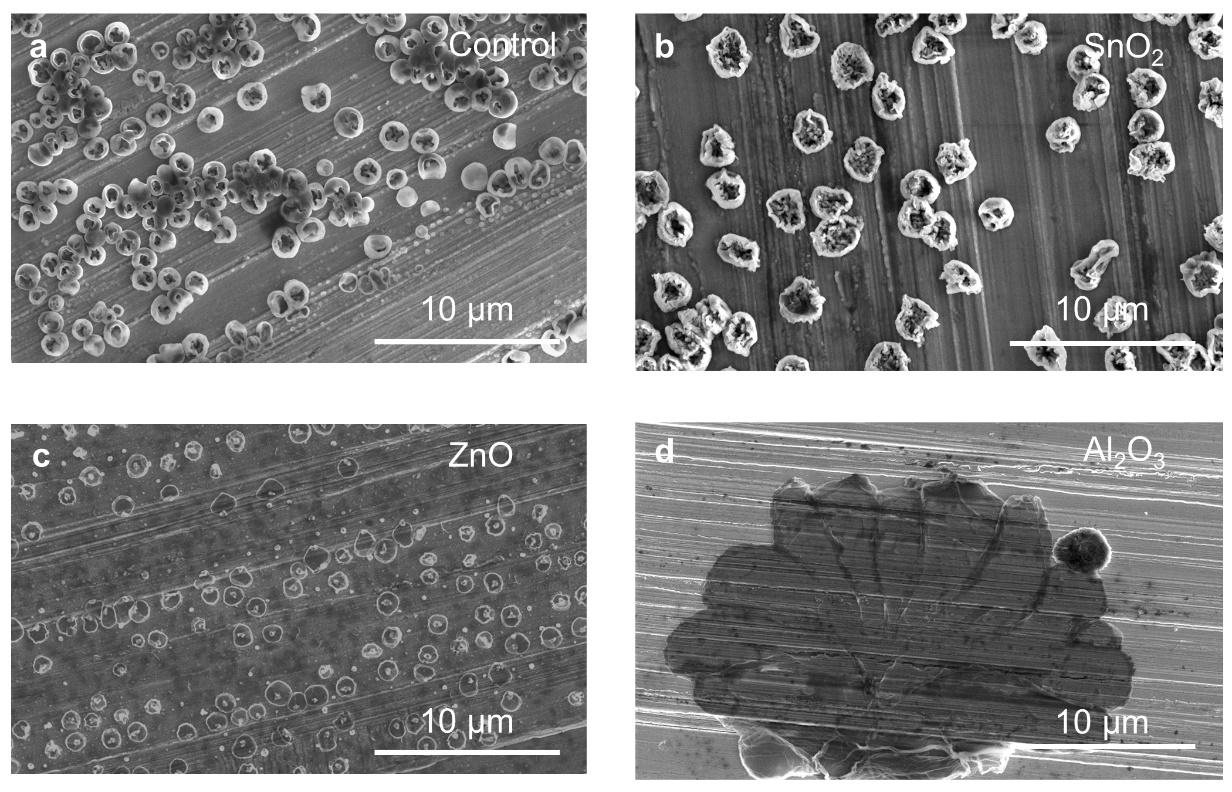


**Supplementary Figure 15.** Top-view SEM images of 0.05 mAh/cm^2^ of lithium plated at 0.02 mA/cm^2^ on all substrates.


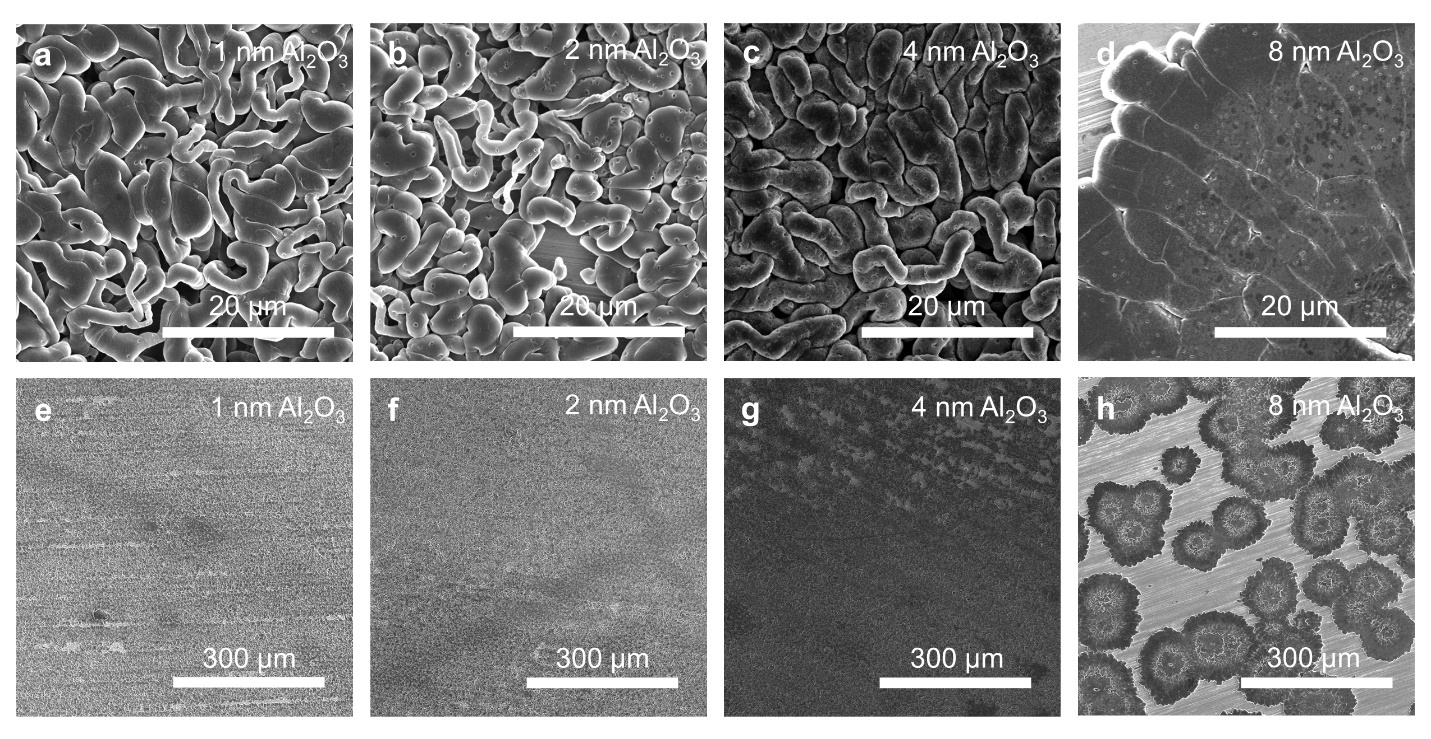


**Supplementary Figure 16.** Top-view SEM images of 1 mAh/cm^2^ of lithium plated at 1 mA/cm^2^ on 1 nm, 2 nm, 4 nm, and 8 nm of Al_2_O_3_-modified copper, respectively, **a-d)** displayed with high magnification, **e-h)** displayed with low magnification.


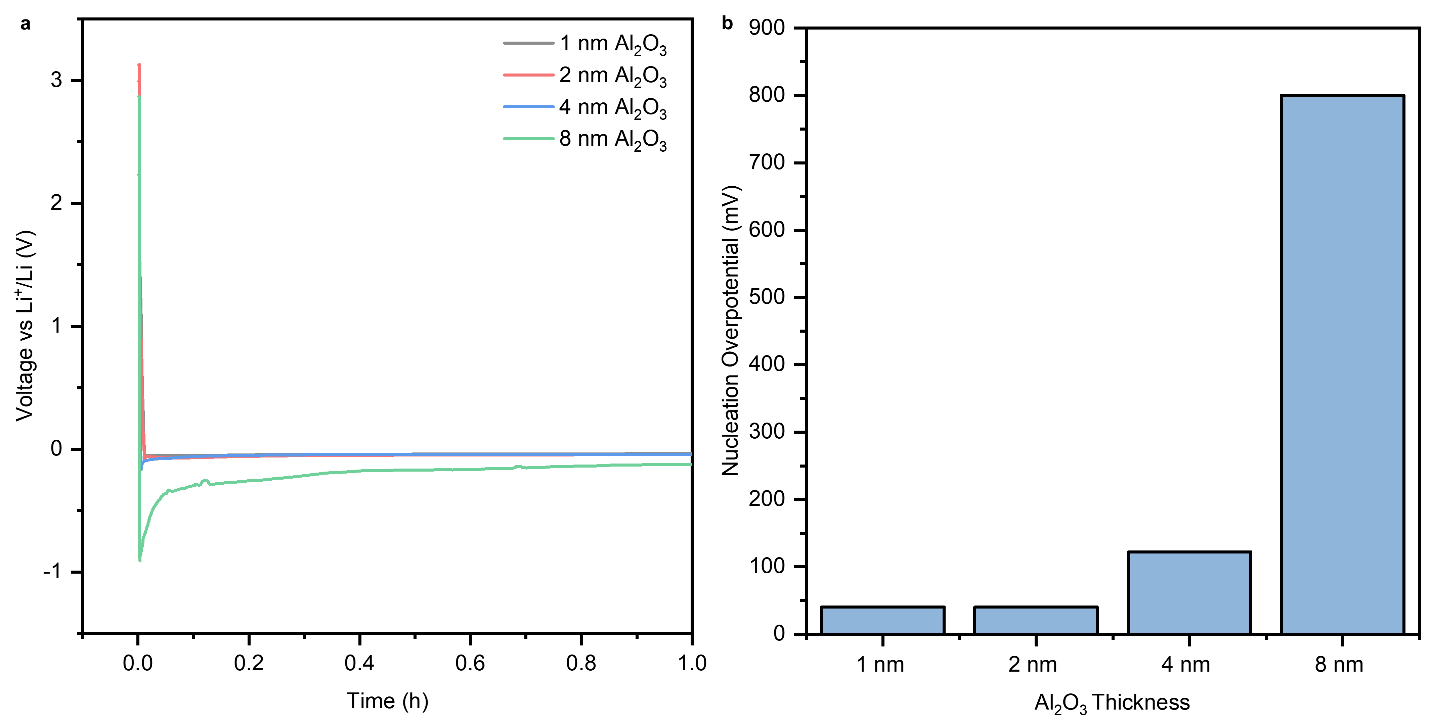


**Supplementary Figure 17. a)** Voltage profiles showing the first cycle of lithium deposition at 1 mA/cm^2^ on 1 nm, 2 nm, 4 nm, and 8 nm Al_2_O_3_-modified substrates. Data labels on the x-axis are omitted for clarity. **b)** Extracted nucleation overpotential of lithium plated at 1 mA/cm^2^ on 1 nm, 2 nm, 4 nm, and 8 nm Al_2_O_3_-modified substrates.


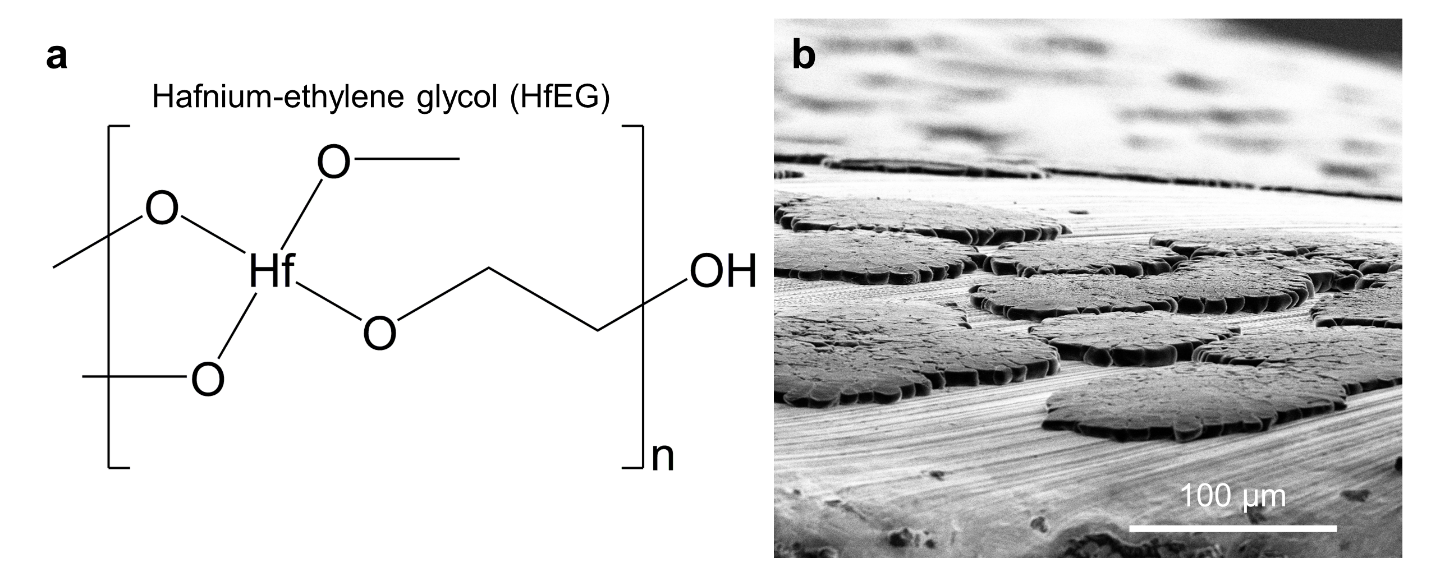


**Supplementary Figure 18. a)** Expected chemical structure of a novel hafnium-ethylene glycol (HfEG) film. **b)** Top-view SEM images of 1 mAh/cm^2^ of lithium plated at 1 mA/cm^2^ on 6 nm HfEG-modified copper substrate.


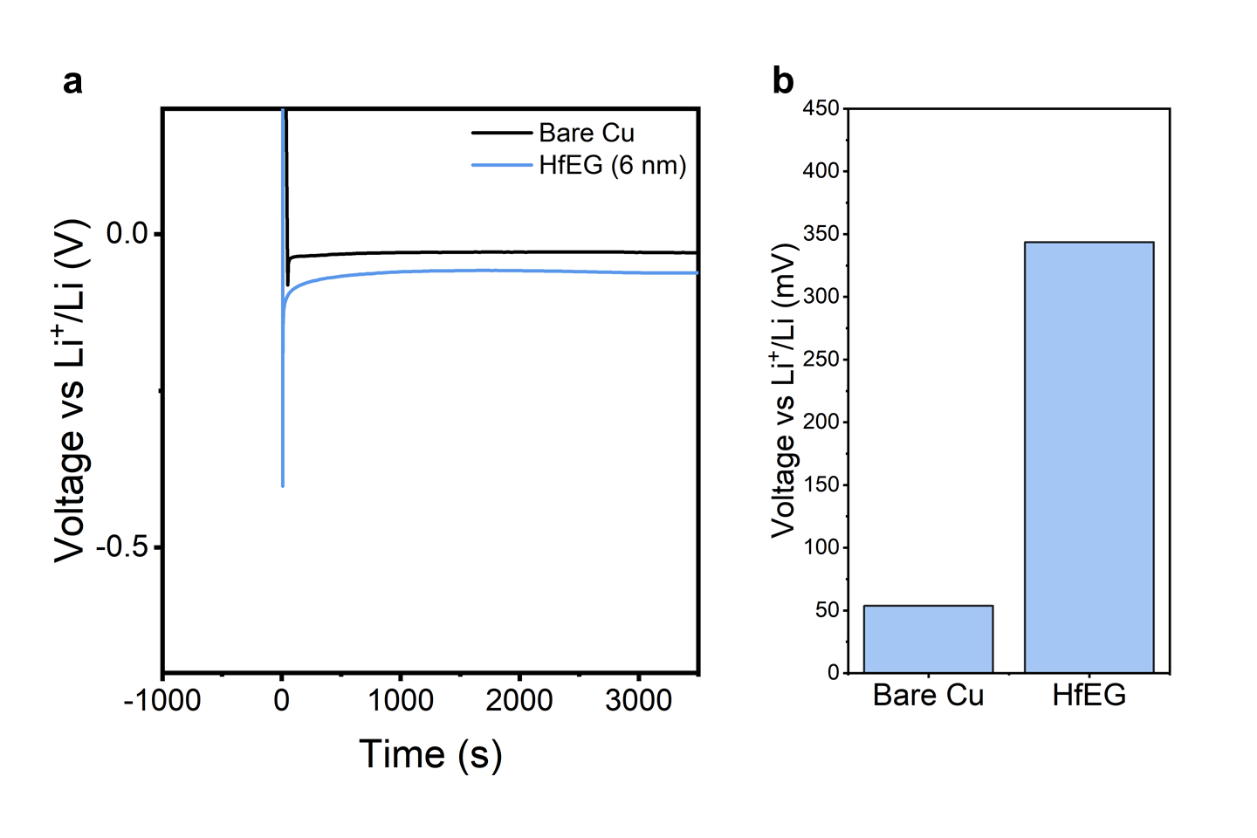


**Supplementary Figure 19. a)** Voltage profiles showing the first cycle of lithium deposition at 1 mA/cm^2^ on bare copper and 6 nm HfEG-modified copper substrate. **b)** Extracted nucleation overpotential of lithium plated at 1 mA/cm^2^ on bare copper and 6 nm HfEG-modified copper substrate.


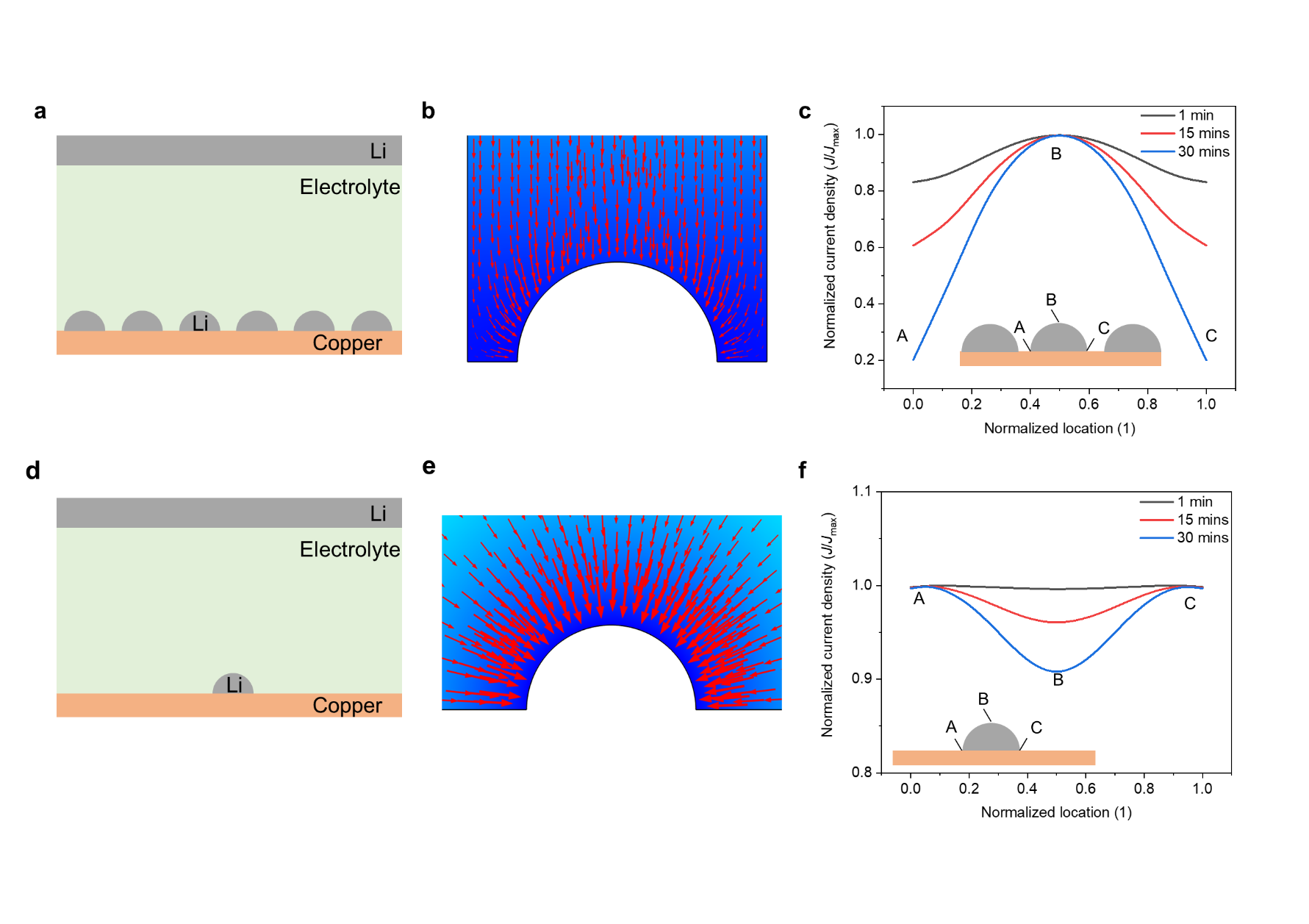


**Supplementary Figure 20.** Finite element COMSOL modeling indicates preference for lateral growth in resistive substrates. **a)** Schematic illustration of conductive substrate as represented in the finite element model, with particles spaced 2.5 µm apart. **b)** Flux of lithium ions towards a representative lithium particle on the conductive substrate in part a, with the thickness of arrows representing current density **c)** Normalized current density as a function of distance across the lithium particle deposited on the conductive substrate shown in figure a. **d)** Schematic illustration of resistive substrate as represented in the finite element model, with particles spaced 200 µm apart. **e)** Flux of lithium ions towards a representative lithium particle on the resistive substrate in part d, with the thickness of arrows representing current density. **f)** Normalized current density as a function of distance across the lithium particle deposited on the resistive substrate shown in figure d. The physical properties used in the COMSOL modeling are shown in **Supplementary Table 2.**


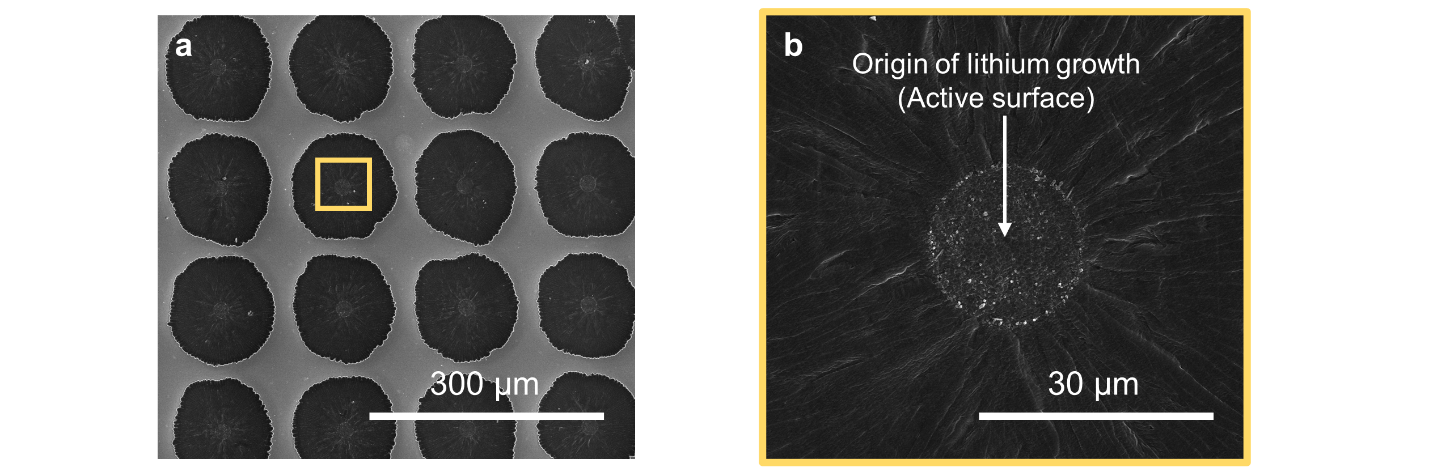


**Supplementary Figure 21.** **a)** Top-view SEM image of lithium deposited atop 50 nm Al_2_O_3_-modified copper substrate patterned with 25 µm sized holes. **b)** Magnified image of the outlined region in panel **a.**


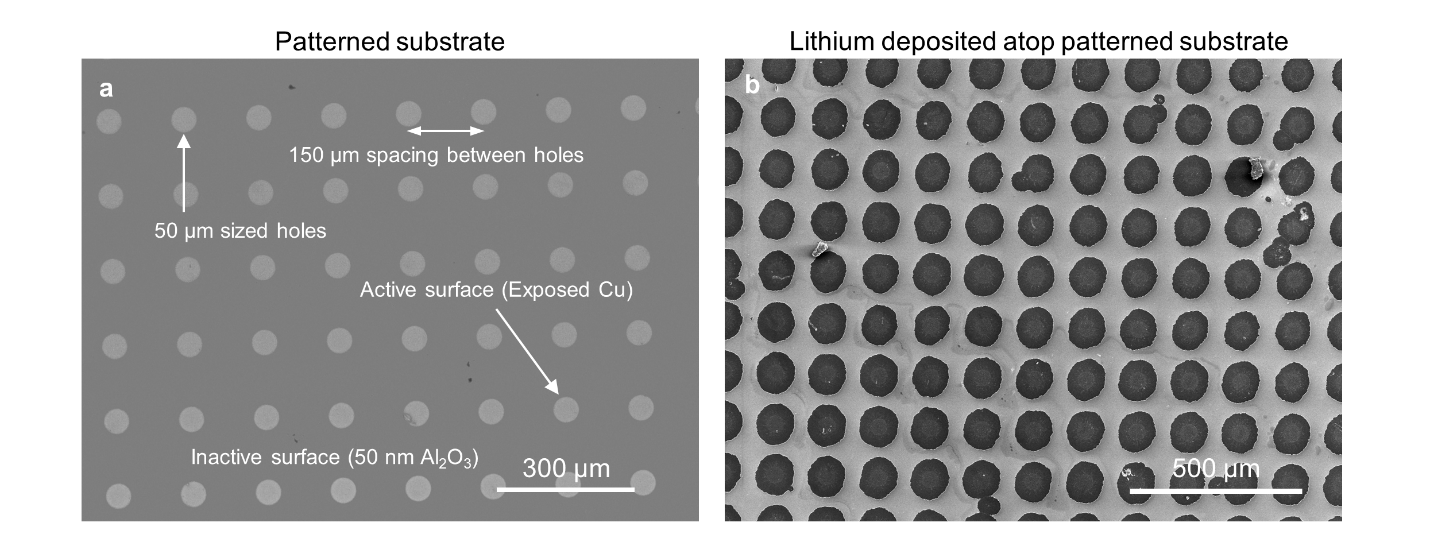


**Supplementary Figure 22.** **a)** Optical image of a 50 nm Al_2_O_3_-modified copper substrate with 50 µm sized holes that expose the underlying copper substrate. **b)** Top-view SEM image of 0.5 mAh/cm^2^ of lithium deposited at 1 mA/cm^2^ atop the patterned substrate shown in panel **a**.

**Supplementary Figure 23.** Anode-free coin cell performance of Cu||NMC 811 cells, using 5 µL of EC/DEC/FEC electrolyte, with a cathode capacity of 4 mAh/cm^2^ and a charge and discharge rate of 1 mA/cm^2^.

**Supplementary note 1**

Sheet resistivity was calculated using:

$$\rho= \frac{V}{I}*4.53 \boldsymbol{1}$$

where R is the sheet resistance, V is the voltage drop across the probe, and I represents applied current. The number, 4.53, is a geometric factor that accounts for thin sheets.

A reference calculation is provided below for SnO_2_:

From Supplementary figure 12 a, at 0.1 mA, the corresponding voltage is 1.6 mV.

From equation 1, sheet resistivity is given as:

$$\rho= \frac{V}{I}*4.53= \frac{1.6 mV}{0.1 mA}*4.53=72.48\frac{\Omega}{sq}$$

**Supplementary note 2**

The diffusion-controlled current-time relationship for the reaction of an electrochemical species at a spherical or hemispherical electrode surface is given as:^5^

$i= \frac{nFA{D_{O}}^{1/2}{C_{O}}^{*}}{\pi^{1/2}t^{1/2}}$ + $\frac{nFAD_{O}{C_{O}}^{*}}{r_{0}}$ **2**

where i is the current, n is the number of electrons transferred during reaction, A is the area of the electrode surface, D_O_ is the diffusion coefficient of the electroactive species, C_O_^*^ is the concentration of the electroactive species in the bulk of the solution phase, t represents time, and r_0_ is the radius of the electrically active site on the electrode.

The first term in the equation represents the Cottrell current (linear diffusion) that would ideally be observed at a planar electrode while the second term represents the contribution of radial diffusion towards the electrode. For UMEs, r_0_ is usually less than 25 µm and as a result, under small timescales, the second term of the equation quickly dominates.^5^

In our system, the active sites (defects and artificial holes) have sizes on the order of UME sizes, suggesting that at steady state, the growth of lithium occurs primarily via radial diffusion of lithium ions via inactive sites towards active sites.

**Supplementary Table 1.** Electrochemical impedance of Li deposited after 50 electrochemical cycles using bare copper and copper modified with ~8 nm of ALD films. R_int_ was obtained from the sum of R2 and R3, with both quantities obtained by fitting the equivalent circuit shown in Supplementary Figure 2. Each fit is carried out using two cell replicates.

| Substrate | R1  (Ohms) | C2  (F) | R2 | C3 | R3 | Q1  (F*s^(a^-^1)) | a1  No unit | R_int_ |
| --- | --- | --- | --- | --- | --- | --- | --- | --- |
| Bare Cu | 23.50  6.97 | 24.07E-06  4.54E-06 | 4.49  2.52 | 7.30E-06  12.84E-06 | 1.99  7.10 | 0.065  0.048 | 0.47  0.43 | **6.48**  **9.62** |
| SnO_2_ (7nm) | 12.50  7.71 | 2.74E-06  12.14E-06 | 2.32  6.42 | 18.96E-06  3.473E-06 | 5.12  2.52 | 0.077  0.047 | 0.46  0.46 | **7.44**  **8.94** |
| ZnO (7 nm) | 10.60  10.40 | 7.45E-06  20.23E-06 | 1.99  4.55 | 27.22E-06  6.17E-06 | 4.16  2.30 | 0.089  0.053 | 0.52  0.46 | **6.15**  **6.85** |
| Al_2_O_3_ (8 nm) | 9.75  7.07 | 24.57E-06  26.57E-06 | 2.49  3.32 | 1.781  7.311E-06 | 0.70  1.57 | 0.106  0.067 | 0.56  0.57 | **3.19**  **4.89** |

**Supplementary Table 2.** Physical properties of Li metal and electrolyte used in the numerical modeling.

| Parameter (unit) | Value | Parameter (unit) | Value |
| --- | --- | --- | --- |
| Diffusion coefficient of Li in electrolyte, *D_Li_* (m^2^⋅s^-1^) | 1×10^-10^ | Initial Li ion concentration in electrolyte, *C_l__*_ini_ (mol⋅m^-3^) | 1000 |
| Electric conductivity of electrolyte, *K_l_* (S⋅m^-1^) | 1.147 | Electrolyte reference concentration, *C_0__*_ref_ (mol⋅m^-3^) | 1000 |
| Transference number of cations, *t_+_* | 0.3 | Density of metallic lithium, ρ (kg/m^3^) | 534 |
| molar activity coefficient of electrolyte, *∂*ln*f* /*∂*ln*C_l_* | 0.43 | Molar mass of metallic lithium, *M*_Li_ (kg/mol) | 0.006941 |
| Reference exchange current density, *i*_0_Ref_ (A/m^2^) | 100 | Cathodic transfer coefficient, *α_c_* | 0.5 |
| Equilibrium potential for Li reaction, *E*_eq_ (V) | 0 | Anodic transfer coefficient, *α_a_* | 0.5 |

**References**

1. Cevher, O. & Akbulut, H. Electrochemical performance of SnO2 and SnO2/MWCNT/graphene composite anodes for Li-Ion batteries. *Acta Phys. Pol. A* **131**, 204–206 (2017).

2. Ferraresi, G. *et al.* SnO2 Model Electrode Cycled in Li-Ion Battery Reveals the Formation of Li2SnO3 and Li8SnO6 Phases through Conversion Reactions. *ACS Appl. Mater. Interfaces* **10**, 8712–8720 (2018).

3. Shen, S., Zhong, W., Huang, X., Lin, Y. & Wang, T. Ordered ZnO/Ni hollow microsphere arrays as anode materials for lithium ion batteries. *Materials (Basel).* **12**, (2019).

4. Liu, Y. *et al.* An Artificial Solid Electrolyte Interphase with High Li-Ion Conductivity, Mechanical Strength, and Flexibility for Stable Lithium Metal Anodes. *Adv. Mater.* **29**, 1–8 (2017).

5. Bard, Allen J. , Faulkner, L. R. *Electrochemical Methods: Fundamentals and Applications*. *2nd ed.; Wiley Global Education: New York* **2**, (2000).
